# Is expected substance type associated with timing of drug checking service utilization?: A cross-sectional study

**DOI:** 10.1186/s12954-021-00514-3

**Published:** 2021-06-27

**Authors:** Tara Beaulieu, Evan Wood, Samuel Tobias, Mark Lysyshyn, Priya Patel, Jennifer Matthews, Lianping Ti

**Affiliations:** 1 British Columbia Centre on Substance Use, 400-1045 Howe St, Vancouver, BC V6Z 2A9 Canada; 2 Graduate Programs in Rehabilitation Sciences, Faculty of Medicine, University of British Columbia, 212-2177 Wesbrook Mall, Vancouver, BC Canada; 3 Department of Medicine, Faculty of Medicine, University of British Columbia, 2775 Laurel Street, Vancouver, BC Canada; 4 School of Population and Public Health, University of British Columbia, 2206 E. Mall, Vancouver, BC Canada; 5 Vancouver Coastal Health Authority, 801-601 West Broadway, Vancouver, BC Canada

**Keywords:** Drug checking, Harm reduction, Stimulant, Opioid, British Columbia

## Abstract

**Background:**

Drug checking is a harm reduction intervention aiming to reduce substance use-related risks by improving drug user knowledge of the composition of unregulated drugs. With increasing fears of fentanyl adulteration in unregulated drugs, this study sought to examine whether the expected type of drug checked (stimulant vs. opioid) was associated with timing of drug checking service utilization (pre-consumption vs. post-consumption).

**Methods:**

Data were derived from drug checking sites in British Columbia between October 31, 2017 and December 31, 2019. Pearson’s Chi-square test was used to examine the relationship between expected sample type (stimulant vs. opioid) and timing of service utilization. Odds ratios (OR) were calculated to assess the strength of this relationship. The Mantel–Haenszel (MH) test was used to adjust for service location.

**Results:**

A total of 3561 unique stimulant and opioid samples were eligible for inclusion, including 691 (19.40%) stimulant samples; and 2222 (62.40%) samples that were tested pre-consumption. Results indicated a positive association between testing stimulant samples and testing pre-consumption (OR = 1.45; 95% CI 1.21–1.73). Regions outside of the epicenter of the province’s drug scene showed a stronger association with testing pre-consumption (OR_MH_ = 2.33; 95% CI 1.51–3.56) than inside the epicenter (OR_MH_ = 1.33; 95% CI 1.09–1.63).

**Conclusion:**

Stimulant samples were more likely to be checked pre-consumption as compared with opioid samples, and stimulant samples were more likely to be tested pre-consumption in regions outside the epicenter of the province’s drug scene. This pattern may reflect a concern for fentanyl-adulterated stimulant drugs.

## Introduction

North America’s overdose epidemic is largely driven by the emergence of illicitly manufactured fentanyl adulteration in the unregulated drug market [1]. Within the last year, there has been growing fear of fentanyl adulteration in stimulant drugs, including in cocaine and methamphetamine [2]. What is particularly alarming about fentanyl adulteration in stimulant drugs is that people who use stimulants may not anticipate such adulterants in their drugs [3], which could lead to higher risk of overdose and death. While the statistics are not entirely conclusive, limited data suggests that fentanyl and related analogues are serving as a key driver in the upsurge of overdose deaths involving stimulants [4].

Drug checking improves user knowledge of drug composition, which has shown to result in the modification of behavior in an effort to reduce harms associated with use [5]. However, studies have also shown that some individuals check their drugs only following an unexpected adverse event [6]. Unfortunately there is a sparsity of literature available to understand the timing of when people choose to check their drugs. With rising fears of fentanyl-adulterated drugs, it may be that people using specific types of drugs are more inclined to check their drugs prior to use. Therefore, the objective of this study was to determine whether the expected type of drug checked (stimulant vs. opioid) was associated with timing of drug checking service utilization (pre-consumption vs. post-consumption) in British Columbia (BC), Canada.

## Methods

Between October 31, 2017 and December 31, 2019, data were derived from point-of-care drug checking services located within harm reduction centres, including in supervised consumption sites, across BC. These programs are described in detail elsewhere [1]. Briefly, the programs employ two drug checking technologies in combination: Fourier transform infrared (FTIR) spectroscopy and fentanyl test strips. These drug checking technologies are commonly used in combination to counterbalance the limitations of each technology on its own [2, 7]. Both technologies produce a result in approximately 5–10 min, and these services are available at no cost during hours of operation that vary by location [2].

For the present analysis, the sample was restricted to samples that were expected by clients to be either a stimulant or an opioid. Expected stimulant samples included: 3-methymethcathinone (3-MMC), 4-fluoroamphetamine (4-FA), 4-methylmethcathinone (4-MMC), amphetamine, cocaine, cocaine and methamphetamine, crack cocaine, methamphetamine, methylphenidate, and methylenedioxymethamphetamine (MDMA). In circumstances where the type of expected stimulant sample was unknown, the sample was coded as “MDMA”. Expected opioid samples included: acetaminophen and codeine (T3), acetaminophen and oxycodone (Percocet), carfentanil, carfentanil and fentanyl, carfentanil and heroin, codeine, fentanyl, fentanyl and heroin, heroin, hydromorphone (Dilaudid), morphine, opium, other, oxycodone, tramadol, 3,4-dichloro-*N*-[(1R,2R)-2-(dimethylamino) cyclohexyl]-*N*-methylbenzamide (U-47700), and unknown opioid (commonly referred to in the community as “down”). These groupings were selected based on consultation with the Drug Wheel and leading toxicology, clinical, and drug checking experts in the area.

The primary outcome of interest was timing of drug checking service utilization, dichotomized as pre-consumption versus post-consumption. The primary explanatory variable was expected sample type, dichotomized as expected stimulant sample versus expected opioid sample. We also considered drug checking service location, dichotomized as Vancouver Downtown Eastside (DTES) neighbourhood (a neighbourhood which includes an open unregulated drug scene and is the epicenter of the overdose epidemic) [8] versus elsewhere in BC.

As a first step, we examined frequency and proportion of expected sample type, stratified by timing of drug checking service utilization. Next, Pearson’s Chi-square test was used to examine the relationship between expected sample type and timing of service utilization. Odds ratios (OR) and 95% confidence intervals (CI) were calculated to assess the strength of this relationship. The Mantel–Haenszel (MH) test was then used to adjust for service location. All analyses were performed using R version 1.1.463 (Foundation for Statistical Computing, Vienna, Austria).

## Results

As shown in Table 1, of the 3561 samples eligible for inclusion, 691 (19.40%) were stimulant samples; 2222 (62.40%) samples were tested pre-consumption. In total, the majority of samples (2999; 84.22%) were tested in Vancouver’s DTES, the epicenter of the province’s drug scene. Results indicated a 1.45 (95% CI 1.21–1.73) higher odds of testing pre-consumption among expected stimulant samples compared to expected opioid samples. In MH analysis, regions outside of the epicenter of the province’s drug scene showed a stronger association with testing pre-consumption (OR_MH_ = 2.33; 95% CI 1.51–3.56) than inside the epicenter (OR_MH_ = 1.33; 95% CI 1.09–1.63).

**Table 1:** Sample characteristics and odds ratios for the relationship between each characteristic and timing of service utilization

Characteristic	Total *n* = 3561, *N* (%)	Timing of drug checking service utilization		Odds ratio (95% CI)
		Pre-consumption, *n* = 2222, *N* (%)	Post-consumption, *n* = 1339, *N* (%)	
Expected sample type				
Opioid	2870 (100.00)	1744 (60.77)	1126 (39.23)	–
Stimulant	691 (100.00)	478 (69.18)	213 (30.82)	1.45 (1.21–1.73)
Drug checking service location				
DTES	2999 (100.00)	1927 (64.25)	1072 (35.75)	1.33 (1.09–1.63)^a^
Elsewhere in BC	562 (100.00)	295 (52.49)	267 (47.51)	2.33 (1.51–3.56)^a^

*CI* confidence interval, *DTES* downtown eastside

^a^Mantel–Haenszel method

## Discussion

Our findings showed that less than two-thirds of samples were tested pre-consumption. The fact that over one-third of samples were tested post-consumption highlights a need to encourage clients to check their drugs prior to consumption—providing an opportunity for immediate harm reduction measures (e.g., reducing dose, using slowly) [6]. Indeed, previous studies have shown safer drug use behavior, including reducing dose, and not using alone (e.g., using supervised consumption sites), following the use of drug checking services [5]. Having said that, it is noteworthy that our findings may be subject to misclassification given that clients checking their drugs may not necessarily be consuming the drug personally (e.g., drug sellers) and therefore, we may be overestimating the number of samples that get checked before use.

Consistent with our primary hypothesis, our findings demonstrated that expected stimulant samples were more likely to be checked pre-consumption as compared with expected opioid samples. Such findings may be attributable to a growing concern for fentanyl-adulterated stimulant drugs in this setting. Alternatively, it may also be that people who use opioids have a high level of trust in their dealer; and for this reason, drug checking is not considered as a priority [9]. Nevertheless, continued efforts should be made to educate people who use stimulants about the risk of fentanyl adulteration, and to ensure that they have access to harm reduction interventions, including those that were previously targeted toward people who use opioids (e.g., naloxone).

That stimulant samples were more likely to be tested pre-consumption in regions outside the epicenter of the province’s drug scene may be partially due to the pervasiveness of fentanyl in the DTES’s unregulated drug supply [10]. There is evidence to suggest that this saturation has led those residing in the DTES to be more accepting of this reality, and less concerned about the potential negative risks associated with ingesting fentanyl-adulterated drugs [10]. To this point, drug checking technologies which provide the ability to approximate quantifications (e.g., FTIR spectroscopy) in addition to qualitative results provided by test strips may be an especially important consideration in settings such as these [1]. This finding provides additional evidence consistent with growing fears of fentanyl adulterated stimulant drugs that have been perpetuated in the media.

There are several limitations to this study. First, data were derived from an anonymous point-of-care program with only few questions asked to make the service as low threshold as possible; thus, we should not exclude the possibility of unmeasured confounders that may potentially bias these findings. Additionally, findings may not be generalizable beyond this setting.

Overall, our findings demonstrate that expected stimulant samples were more likely to be tested prior to consumption as compared with expected opioid samples, and that this was more likely to occur in regions outside the epicenter of the province’s drug scene—perhaps a reflection of growing fears of fentanyl-adulterated stimulant drugs, which may vary by region. This exploratory study, one of the first contributions to provide insights into whether expected type of drug checked is associated with timing of drug checking service utilization, opens potential avenues for future research to examine this issue more rigorously.

## Data Availability

The dataset supporting the conclusions of this article is available on the British Columbia Centre on Substance Use website, https://drugcheckingbc.ca/results/.

## Abbreviations

BC: British Columbia
CI: Confidence interval
DTES: Downtown Eastside
FTIR: Fourier transform infrared
MH: Mantel–Haenszel
MDMA: Methylenedioxymethamphetamine
OR: Odds ratios
U-47700: 3,4-Dichloro-*N*-[(1R,2R)-2-(dimethylamino) cyclohexyl]-*N*-methylbenzamide
3-MMC: Methymethcathinone
4-FA: 4-Fluoroamphetamine
4-MMC: 4-Methylmethcathinone
